# Pulp Response following Direct Pulp Capping with Dentin Adhesives and Mineral Trioxide Aggregate; An Animal Study 

**DOI:** 10.22037/iej.2017.44

**Published:** 2017

**Authors:** Ali Akhavan, Farahnaz Arbabzadeh, Majid Bouzari, Sayed Mohammad Razavi, Amin Davoudi

**Affiliations:** a *Dental Materials Research Center, Department of Endodontics, Dental School, Isfahan University of Medical Sciences, Isfahan, Iran; *; b * Dental Research Center, Department of Operative Dentistry, Dental School, Isfahan University of Medical Sciences, Isfahan, Iran; *; c * Department of Biology, Faculty of Sciences, University of Isfahan, Isfahan, Iran; *; d *Dental Implants Research Center, Department of Oral and Maxillofacial Pathology, Dental School, Isfahan University of Medical Sciences, Isfahan, Iran; *; e *Dental Students' Research Committee, Department of Prosthodontics, Dental School, Isfahan University of Medical Sciences, Isfahan, Iran*

**Keywords:** Dentin Adhesive Systems, Direct Pulp Capping, Mineral Trioxide Aggregate

## Abstract

**Introduction:**

Pulp vitality and its continuous dentin prodution are essential for long-term success of direct pulp capping (DPC). The aim of present study was to evaluate the histopathological response of the canine pulp following DPC using either different dentin adhesive resins (DAR), calcium hydroxide (CH) or mineral trioxide aggregate (MTA).

**Methods and Materials:**

DPC was done on 72 dog’s teeth using 6 types of dental materials (*n*=12) (4 types of DAR, white MTA and CH). Therefore, six healthy dogs were anesthetized and 2 teeth from each dog were allocated to either type of mentioned DPC agents. The dental pulps were exposed mechanically by drilling in the center of class V cavities. The different types of capping materials included DARS (Clearfil S3 Bond, Optibond FL, Single Bond and Clearfil SE Bond), white MTA and CH. After 7, 21 and 63 days, two dogs were euthanized in each interval. Microscopic evaluations were done according to following criteria: intensity of inflammation, presence of necrosis and formation of hard tissue. The recorded data were analyzed by the Kruskal-Wallis, Friedman, Cochran’s and Fisher’s exact tests using SPSS software version 12 at significant level of 0.05.

**Results:**

No significant differences were found regarding necrosis among DPC materials (*P*>0.05). However, MTA caused higher amount of hard tissue formation after 63 days in comparison with 21 days.

**Conclusion:**

MTA provided the highest degree of hard tissue formation after 63 days. However, further studies should be performed for administering a definitive material.

## Introduction

Direct pulp capping (DPC) is a treatment protocol for reversible pulp injuries which aims to maintain function and vitality of the pulp [1]. The pulp might be injured by either caries or restorative procedures. Vital and functional dentin is needed beneath the injured site for long term success of DPC [2]. Therefore, pulp capping materials may influence the success rate of DPC [3]. An ideal capping agent for DPC should have the capability of inhibiting inflammation, bonding to dentin, microleakage prevention, easy to facilitate and inducing formation of dental bridge [4]. In addition, materials which are used in DPC should be able to stimulate forming of tertiary dentin. Calcium Hydroxide (CH) is one of the most common agents which is widely used in DPC. Although CH causes formation of reparative dentin, it causes a necrotic layer due to its high alkalinity (pH=12) which may lead to inflammatory pulp responses [5]. Due to physical limitations of CH such as lack of adhesion to dentin, dissolution in internal fluids and failure of resistance under tooth flexure, researchers have explored for new materials [6]. Dentin adhesive resin systems (DAR) are among materials which have been proposed as substitutes to CH [7-9]. DAR have lower pH and cause less aggravations to the pulp. Additionally, it has been shown that self-etching resins have antimicrobial activities and alleviated gap formation and microleakage. The most important fact in application of DAR is their proper sealing ability in dentinal margins [10].

**Figure 1:**
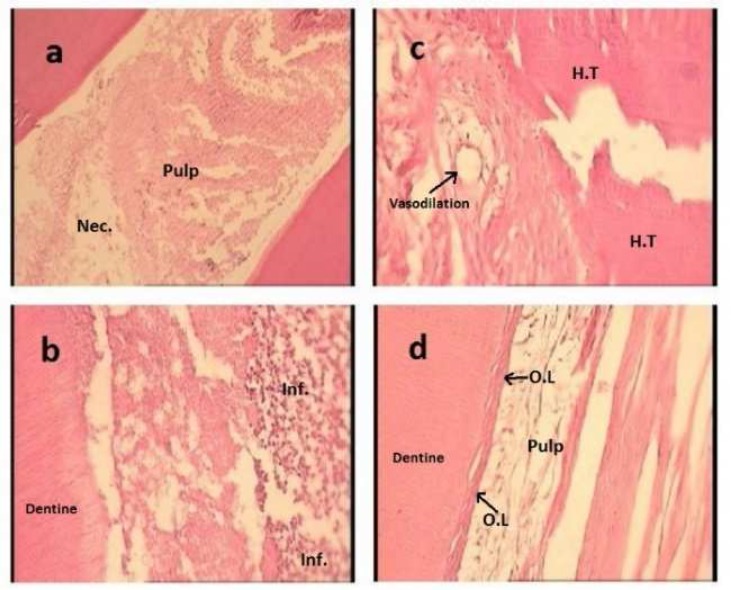
Necrosis caused by CSE after 7 days (×100); *A)* inflammation response (type 3) to OBF after 7 days (×400); *B)* hard tissue formation which was caused by MTA after 63 days (×400); *C)* odontoblastic layer formation which was induced by MTA after 63 days (×400); *D)* The abbreviations stand for: necrosis (Nec.), hard tissue formation (H.T), inflammation (Inf.), odontoblastic layer (O.L

Nowadays, MTA has attracted attentions as substitute for materials such as CH [10-13]. It characterizes successful clinical bioactivity, and it has been administered for sealing exposed pulps, repairing perforations, apexification and retrograde filling [9, 14]. Nevertheless, there has been controversy among studies in which CH and MTA were used as a DPC agents. CH might be a successful DPC agent when used in human primary teeth [15]. MTA revealed better results in maintaining pulp vitality after DPC both clinically and histologically [16]. 

During the formation of reparative dentin, primary odontoblasts (which were destroyed at the exposed site) would be replaced by new differentiated odontoblast-like cells which were differentiated from stem cells [1]. In order to assess histopathological response of the pulp to different pulp capping agents, the presence or formation of following items should be evaluated: inflammation, hard tissue, odontoblast cell layer, dentinal bridge and necrotic layer [1, 16]. 

As the DAR have shown positive properties in inhibiting gap formation, preventing microleakage, absence of pulp irritation due to dentinal fluid movement; and the calcifying properties of MTA and CH, the present study aimed to investigate the pulpal response to DAR, CH and MTA in healthy dog’s teeth after DPC.

## Materials and Methods

Ethical approval was granted by the Torabinejad Dental Research Center, Isfahan University of Medical Sciences, Isfahan, Iran (ID No.: 185261). All procedures were conducted strictly in accordance with ethical standards and with the last update of the Helsinki Declaration. The maintenance and care of the animals complied with the ethical guidelines of the Torabinejad Dental Research Center.

This prospective study, with descriptive and analytical design, was conducted on total of 72 teeth from six mature and healthy dogs (12 teeth in each dog). The teeth of each dog were divided into 6 groups (*n*=2) (4 groups for DRA, 1 group for MTA, and 1 group for CH) (Table 1). 

The dogs were anesthetized, with 20 mg/kg of Ketamine HCL (Alfasan, Woerden, Netherland) and 12 mg/kg of Xylazine (Alfasan, Woerden, Netherland), under supervision of a veterinarian at Torabinejad Dental Research Center. The oral cavity was disinfected by rinsing with 0.2% chlorhexidine (Shahdaru Labratories, Tehran, Iran). After local anesthesia infiltration, with 1.8 mL of 2% lidocaine with 1:100000 epinephrine (Xylocaine; Dentsply Pharmaceuticals Inc, York, PA, USA), the premolars; canines; first, second and third incisors (twelve teeth in total) were sealed and polished with rubber cups in each dog. Class V cavities with 2.5 mm width, 3 mm length and 1.5 to 2 mm depth were made on buccal surfaces by carbide burs (Teeskavan, Tehran, Iran) parallel to CEJ. Each cavity was prepared by a new bur to avoid heat trauma to the pulp. In the next step, pulp exposure was executed by drilling the center of class V cavities and using high speed round burs (Teeskavan, Tehran, Iran) with approximate diameter of 0.5 mm. The size of exposure point was about 0.8 to 1 mm and bleeding was controlled by mild pressure of cotton pellets. The exposure points were rinsed with sterilized normal saline, and dried using cotton pellets. In eight teeth of each dog DPC was done with the following types of DAR, separately: Clearfil S3 Bond (CS3) (Kuraray, Osaka, Japan) (*n*=2 in each dog and *n*=12 in total) Optibond FL (OBF) (Kerr, Orange, CA, USA) (*n*=2 in each dog and *n*=12 in total), Single Bond (SB) (3M ESPE, MN, USA) (*n*=2 in each dog and *n*=12 in total), Clearfil SE Bond (CSE) (Kuraray, Tokyo, Japan) (*n*=2 in each dog and *n*=12 in total). The manipulation of DAR was done based on manufacturer instructions. Then, the teeth were restored with nano-filled composite resins (Filtek Supreme XT, 3M ESPE, MN, USA), incrementally. 

In the last four teeth in each dog, DPC was done using with white MTA (Pro-Root MTA Densply, Tulsa, USA) and CH (Dycal, Dentsply, Germany). They were lined with light-cured Glass Ionomer (Grandio, Voco, Germany) and then restored with nano-filled composite resin, incrementally. 

The application of DAR and restorative procedures were conveyed by a dental operative and restorative specialist. Also, the manipulation of MTA and CH was conducted by an endodontist.

After 7, 21 and 63, two dogs were euthanized in each interval based on vital perfusion method with 10% formalin solution (Merck, Germany). The decalcification procedure was followed by 10% formic acid solution for 20 days, dehydration with grated alcohols and embedding in paraffin. All specimens were sectioned through longitudinal axis of the tooth. Slides were prepared and samples were stained with Hematoxylin and Eosin (H&E) for blind microscopic evaluations by an oral and maxillofacial pathologist. Finally, the following criteria were observed: intensity and type of inflammation, presence or absence of necrosis and formation or lack of hard tissue. The recorded data were graded based on qualitative ranking system [17, 18] (Table 2) and the obtained data were analyzed by the Kruskal-Wallis, Friedman, Cochran’s and Fisher’s exact tests using Statistical Package for Social Science (SPSS, version 12.0, SPSS, Chicago, IL, USA) at significance level of 0.05. 

**Table 1:** The sample size and number of allocated teeth for each group

	**SB**	**CS3**	**OBF**	**CSE**	**CH**	**MTA**		**Total**	
**Dog 1**	2	2	2	2	2		2		12	
**Dog 2**	2	2	2	2	2		2		12	
**Dog 3**	2	2	2	2	2		2		12	
**Dog 4**	2	2	2	2	2		2		12	
**Dog 5**	2	2	2	2	2		2		12	
**Dog 6**	2	2	2	2	2		2		12	
**Total**	12	12	12	12	12		12		72	

**Table 2:** The criteria for ranging pulp response to different DPC materials

**Inflammation Intensity**	**Hard tissue formation**		**Necrosis**
	**Odontoblastic cell layer**	**Dentinal or hard tissue bridge**	
**0) Without inflammation (No inflammatory cells) **	0) No odontoblastic cell layer	0) No evidence of hard tissue formation	0) Without
**1) Mild inflammation (< 30 inflammatory cells)**			
**2) Moderate inflammation (30-60 inflammatory cells)**	1) Presence of odontoblastic cell layer	1) Presence of hard tissue formation	1) Signs of necrosis
**3) Severe inflammation (> 60 inflammatory cells)**			

**Table 3:** The distribution and number of samples which showed different pulpal responses to the DPC materials

**Days**	**Pulp response**	**SB**	**CS3**	**OBF**	**CSE**	**CH**	**MTA**	
**7**	**Inflammation**	1	1	2	1	1	1
	**Hard tissue**	2	2	0	1	1	1
	**Odontoblastic layer**	2	2	1	1	2	1
	**Necrosis**	0	0	0	1	0	0
**21**	**Inflammation**	1	2	4	3	3	3
	**Hard tissue**	2	2	3	2	1	0
	**Odontoblastic layer**	4	1	3	2	3	3
	**Necrosis**	0	0	0	0	0	0
**63**	**Inflammation**	4	3	4	4	4	1
	**Hard tissue**	1	1	2	0	2	4
	**Odontoblastic layer**	1	2	2	0	2	4
	**Necrosis**	2	0	1	1	0	0

**Table 4.** The comparison (*P*-values) of different pulpal response in different intervals which were caused by different DPC materials

**Pulp response**	**days**	**SB**	**CS3**	**OBF**	**CSE**	**CH**	**MTA**
**Necrosis**	7 and 21	0.43	0.40	0.40	0.60	0.66	0.66
	7 and 63	0.40	0.55	0.66	0.33	0.40	0.60
	21 and 63	0.21	0.33	0.50	0.50	0.50	0.50
**Hard tissue**	7 and 21	0.40	0.40	0.20	0.80	0.60	0.33
	7 and 63	0.20	0.20	0.40	0.33	0.60	0.33
	21 and 63	0.50	0.50	0.50	0.41	0.78	0.014
**Odontoblastic layer**	7 and 21	0.60	0.20	0.60	0.80	0.66	0.60
	7 and 63	0.20	0.40	0.80	0.33	0.40	0.33
	21 and 63	0.71	0.50	0.50	0.24	0.50	0.50

## Results

Following results were obtained from the qualitative values of pulp responses to DPC after 7, 21 and 63 days (Table 3). 

After 7 days, CSE caused necrosis in one sample (Figure 1A); OBF induced inflammation in two samples (Figure 1B); SB and CS3 inducted formation of hard tissue and odontoblast layer in two samples. 

After 21 days, inflammation was observed due to DPC by OBF in four teeth; and SB stimulated odontoblastic layer generation in four samples. 

The results represented that MTA caused the lowest inflammation (*n*=4), the highest amounts of hard tissue (*n*=4) (Figure 1C) and odontoblastic layer formations (*n*=4) (Figure 1D) after 63 days.

Based on the results of Fisher’s Exact test (Table 4), no case of necrosis was found among DPC materials (*P*>0.05). However, MTA caused higher amount of hard tissue formation after 63 days in comparison with 21 days (*P*=0.014); however, according to the Dunn-Bonferroni test this level might not be significant.

In intra-group comparison, Friedman test revealed no significant differences in the intensity and severity of inflammation during all time intervals (*P*>0.05). 

## Discussion

Present study investigated the pulpal response of healthy dog teeth after DPC to different DPC agents naming DAR, MTA and CH after three different intervals. Histopathological changes were evaluated in terms of necrosis, type and intensity of inflammation, regenerations of hard tissue and odontoblastic layer. Based on the results, no significant difference was found among different DPC materials, except for MTA which showed significantly the highest degree of hard tissue formation after 63 days (Figure 1C). MTA can promote cells to form hard tissues and create an environment for mineralization [1, 2]. Additionally, this material releases CH and interacts with materials containing phosphates and precipitates apatite [19]. These properties of MTA correspond to present study. Silva *et al. *[20] investigated pulpal tissue response to SB and CH. They indicated that pulpal responses to both agents were identical after 30 days, which is in accordance with the results of present study. Dammaschke *et al.* [21] found the same results, as well.

Variable responses, ranging from mild to severe inflammation, were recorded in this study. In general, dentinal bridge formation was observed in a DPC group done by CH. However, CH causes inflammatory cells infiltration, partial necrosis and partial to complete hard tissue formation [22].

The most important issue in application of DAR is the sealing capability which can be achieved in dentinal margins. When a decay is so deep, exposure and perforation might be inevitable in limited locations of the pulp. Thus, proper hybridized dentin-adhesive interface can seal both dentin and pulp and protect the injured regions from further damages and infections, effectively. This shield allows complete healing through intrinsic dentin-pulp complex capabilities [6, 10-12]. The results of present study were in contrast with these mentioned findings. Hard tissue was not observed for SB group in the present study. Softening and separation might be influenced by acid-etching technique prior to use of DAR. This fact may contaminate bonding agents and increase the possibility of microleakage and decrease of survival rates, consequently [23]. However, CH group showed no significant difference in comparison with SB group in other phenomena. In one study, pulpal tissue inflammatory response showed no significant difference after DPC by CSE and CH after 7 days [24].

Although, mentioned findings were in agreement with current results, the amount of dentin bridge formation was lower for CH samples in present study. Lu Y *et al*. [25] declared that dentin preparation potential of CSE was lower than CH, which also disagrees with present results. Self-etching primers react with the dentin and enamel by their soluble acidic monomers. So, they cannot be rinsed and do not interfere with the movement of dentin tubular fluids. Also, based on hydrodynamic theory, it is possible that the self-etching primers would not aggravate pulpal condition [26]. Data regarding histopathological response of pulpal tissue to DAR are limited, contradicted and not reliable for suggesting a definitive performance of different capping agents. on the other hand, some researches support that some other dental materials, such as CEM cement, might provide promising biological results as DPC agent [27-29]. 

## Conclusion

Based on the results, MTA provided the highest degree of hard tissue formation after 63 days, which proves it to be useful for long-term administration. But, further studies should be performed on other dental materials with larger number of samples, more intervals, and preferably on human teeth.
